# Validation and Refinement of the Sense of Coherence Scale for a French Population: Observational Study

**DOI:** 10.2196/50284

**Published:** 2024-07-16

**Authors:** Paul Sebo, Benoit Tudrej, Augustin Bernard, Bruno Delaunay, Alexandra Dupuy, Claire Malavergne, Hubert Maisonneuve

**Affiliations:** 1 Institut universitaire de Médecine de Famille et de l'Enfance (University Institute for Primary Care) University of Geneva Geneva Switzerland; 2 University College of General Medicine University Claude Bernard Lyon 1 Lyon France

**Keywords:** French, sense of coherence, salutogenesis, SOC, Sense of Coherence scale, validation, validscale, well-being, promoting, resilience, validity, reliability, primary care patients, manageability

## Abstract

**Background:**

Salutogenesis focuses on understanding the factors that contribute to positive health outcomes. At the core of the model lies the sense of coherence (SOC), which plays a crucial role in promoting well-being and resilience.

**Objective:**

Using the *validscale* Stata command, we aimed to assess the psychometric properties of the French version of the 3-dimension 13-item SOC questionnaire (SOC-13), encompassing the comprehensibility, manageability, and meaningfulness dimensions. We also aimed to determine if a refined scale, assessed through this method, exhibits superior psychometric properties compared to the SOC-13.

**Methods:**

A sample of 880 consecutive primary care patients recruited from 35 French practices were asked to complete the SOC-13. We tested for internal consistency and scalability using the Cronbach α and Loevinger H coefficients, respectively, and we tested for construct validity using confirmatory factor analysis and goodness-of-fit indices (root mean square error of approximation [RMSEA] and comparative fit index [CFI]).

**Results:**

Of the 880 eligible patients, 804 (91.4%) agreed to participate (n=527, 65.6% women; median age 51 years). Cronbach α and Loevinger H coefficients for the SOC-13 were all <0.70 and <0.30, respectively, indicating poor internal consistency and poor scalability (0.64 and 0.29 for comprehensibility, 0.56 and 0.26 for manageability, and 0.46 and 0.17 for meaningfulness, respectively). The RMSEA and CFI were >0.06 (0.09) and <0.90 (0.83), respectively, indicating a poor fit. By contrast, the psychometric properties of a unidimensional 8-item version of the SOC questionnaire (SOC-8) were excellent (Cronbach α=0.82, Loevinger H=0.38, RMSEA=0.05, and CFI=0.97).

**Conclusions:**

The psychometric properties of the 3-dimension SOC-13 were poor, unlike the unidimensional SOC-8. A questionnaire built only with these 8 items could be a good candidate to measure the SOC. However, further validation studies are needed before recommending its use in research.

## Introduction

Salutogenesis, a concept developed by Aaron Antonovsky, represents a paradigm shift in health research as an approach focusing on understanding the factors contributing to positive health outcomes rather than merely concentrating on disease prevention [1,2]. At the core of Antonovsky’s salutogenic model lies the concept of the sense of coherence (SOC), a multifaceted concept reflecting an individual’s capacity to comprehend, manage, and find meaning in the world around them, influencing their ability to cope with stressors and maintain positive well-being [3]. The three interrelated dimensions of SOC include comprehensibility (perceiving the world as ordered/predictable), manageability (belief in coping effectively with stressors), and meaningfulness (finding purpose/motivation in life). A strong SOC fosters a cognitive orientation that enables individuals to perceive their environment as structured/predictable, facilitating a greater understanding of the challenges they encounter. Moreover, the belief in one’s ability to manage stressors effectively empowers individuals to approach difficulties with confidence/resilience. The sense of meaningfulness, derived from finding purpose/motivation in life, further contributes to an individual’s adaptive capacity in the face of stressors. The SOC theory also introduces a unidimensional model that provides a consolidated measure, aiding in a quicker clinical assessment of an individual’s overall SOC. The choice between the 3-dimension and unidimensional models depends on assessment goals and the required depth of information.

The SOC theory has gained considerable attention in health research, with numerous studies exploring its applicability/implications [3,4]. Researchers typically use the 13-item questionnaire (SOC-13) to assess an individual’s level of coherence and its association with various health-related outcomes [4,5]. The questionnaire has been translated into several languages, including French. However, to our knowledge, the French version did not go through validation procedures. A relatively old population-based study evaluated a French version of the SOC-13 scale modified by the authors [6]. This questionnaire (not provided in their article) showed satisfactory internal consistency but only for the unidimensional model, whereas the validity was insufficient.

Given the lack of information on the validity/reliability of the French SOC-13, we aimed to assess its psychometric properties in primary care patients, ensuring its appropriateness/effectiveness for assessing SOC in French-speaking patients. If the psychometric properties of this scale were found to be insufficient, a secondary objective was to develop an alternative version that would be more valid/reliable than the SOC-13. By selecting primary care patients as the target, we explored the SOC concept in a real-world, patient-centered setting, recognizing implications for interventions and the broader relevance to salutogenesis.

## Methods

### Study Setting

This observational study was performed with primary care patients in France during 2023. We used a professional register of primary care physicians in the Rhône-Alpes region of France and randomly selected 200 physicians using computer-generated random numbers. Five research assistants contacted each selected physician via email until the target number of physicians (n=35) was attained. In case of refusal or no response after 3 reminders, the next practice on the list was contacted. A sample of 880 consecutive patients recruited from these practices (20-25 patients per practice) were asked to complete the French SOC-13 in the waiting room. Eligible participants were nonurgent, French-speaking, adult patients capable of understanding the study.

### Ethical Considerations

The study was approved by the Research Ethics Committee of the University College of General Practice (Lyon) (project ID IRB 2023-01-03-01). Informed consent was obtained from all participants and their ability to opt out was ensured. Privacy/confidentiality were maintained through anonymized data.

### SOC-13 Scale

The SOC-13 questionnaire has three components: items 2, 6, 8, 9, and 11 are related to comprehensibility; items 3, 5, 10, and 13 are related to manageability; and items 1, 4, 7, and 12 are related to meaningfulness. The questions are rated on a 7-point Likert scale so that the total score ranges from 13 to 91. The coding for items 1, 2, 3, and 7 is reversed. We summarized the 3 subscores and the total score using the median (IQR).

### Validation of the French Version of the 3-Dimension SOC-13 and Development of the Unidimensional 8-Item SOC Questionnaire

We used the *validscale* command [7] in Stata to assess the psychometric properties of the SOC-13 using classical test theory [8]. We assessed both the 3-dimension and unidimensional models with this approach. We tested for internal consistency and scalability using the Cronbach α and Loevinger H coefficients, respectively. A minimum value of 0.70 for Cronbach α and of 0.30 for Loevinger H were considered acceptable [9,10]. We tested for construct validity using confirmatory factor analysis and goodness-of-fit indices. To assess the adequacy of the statistical model, we used the root mean square error of approximation (RMSEA) and the comparative fit index (CFI). These indices evaluate the agreement between the observed and expected data according to the specified model. An RMSEA<0.06 and a CFI>0.90 are generally considered to indicate that the model is a good fit [11]. We used the *convdiv* option to assess convergent/divergent validities through examination of a correlation matrix [7].

We also developed a shorter questionnaire in French that is potentially more reliable/valid and easier to use in primary care than the SOC-13. We removed all problematic items from the SOC-13 by examining the Cronbach α values obtained for each removed item, while keeping at least 2 questions per dimension. This questionnaire consisted of 8 items (items 6, 8, 9, and 11 for comprehensibility; items 10 and 13 for manageability; and items 4 and 12 for meaningfulness).

The French versions of the SOC-13 and the new unidimensional 8-item SOC scale (SOC-8) are provided in Multimedia Appendix 1. Following published guidelines, we targeted a minimum of 500 participants, achieving a “very good” sample size, with a responder-to-item ratio exceeding 20:1 [12]. All analyses were performed with Stata 15.1.

## Results

A total of 804 participants agreed to take part in the study (participation rate=91.4%), 65.6% of whom were women (n=527). The median age of the participants was 51 (IQR 30, range 20-93) years. Depending on the item, between 787 and 793 participants responded to the SOC-13 questions. The median score was 23 (IQR 8, range 5-35) for comprehensibility, 18 (IQR 6, range 4-28) for manageability, 21 (IQR 6, range 8-28) for meaningfulness, and 62 (IQR 16, range 30-89) for the total score.

Internal consistency and scalability were not sufficient for the 3-dimension model. Cronbach α and Loevinger H coefficients were all <0.70 and <0.30, respectively (0.64 and 0.29 for comprehensibility, 0.56 and 0.26 for manageability, and 0.46 and 0.17 for meaningfulness, respectively). Table 1 shows the proportion of missing data for each item, the distribution of item responses, and the Loevinger H and Cronbach α coefficients obtained by omitting each item. Loevinger H coefficients were <0.30 for 9 of the 13 items.

The confirmatory factor analysis, goodness-of-fit indices, and correlation matrix are shown in Table 2. The RMSEA and CFI were 0.09 and 0.83, respectively, indicating a poor fit. Only 4 items had a correlation coefficient with the score of their own dimension >0.40 (indicating lack of convergent validity) and only 5 items had a correlation coefficient with the score of their own dimension greater than those computed with other scores (indicating lack of divergent validity).

**Table 1:** Distribution of item responses, internal consistency, and scalability of the French versions of the 3-dimension 13-item and unidimensional 8-item sense of coherence (SOC) scales.

Scales and items					Missing data, %			Patients, n			Response category, %																						Cronbach α^a^				Loevinger H
											1			2			3			4			5			6			7								
**SOC-13**																																					
	**Dimension 1: comprehensibility**																																				
		Item 2	1.37			793			8.45			19.29			30.77			22.32			10.09			6.43			2.65			0.71				0.10			
		Item 6	1.37			793			3.28			7.44			10.34			16.27			19.17			27.49			16.02			0.53				0.35			
		Item 8	1.37			793			3.28			7.06			12.11			19.55			15.51			25.35			17.15			0.51				0.37			
		Item 9	2.11			787			2.03			5.84			11.56			18.17			14.49			25.54			22.36			0.55				0.33			
		Item 11	2.11			787			3.30			8.51			12.33			22.24			19.44			23.38			10.80			0.61				0.26			
	**Dimension 2: manageability**																																				
		Item 3	1.74			790			8.73			16.58			29.49			18.10			12.66			11.27			3.16			0.57				0.20			
		Item 5	1.37			793			3.40			5.80			11.98			20.18			17.40			24.46			16.77			0.50				0.25			
		Item 10	1.87			789			3.17			11.15			15.72			17.49			13.94			20.66			17.87			0.43				0.30			
		Item 13	2.11			787			2.54			6.35			10.67			14.23			16.90			31.77			17.53			0.46				0.29			
	**Dimension 3: meaningfulness**																																				
		Item 1	1.37			793			5.04			5.42			11.73			14.75			14.00			25.98			23.08			0.55				0.06			
		Item 4	1.37			793			2.02			1.77			2.27			13.11			20.81			33.80			26.23			0.31				0.22			
		Item 7	1.49			792			1.39			4.17			6.82			20.45			22.35			30.56			14.27			0.34				0.21			
		Item 12	1.99			788			4.31			5.46			9.14			13.07			19.04			32.11			16.88			0.32				0.21			
**SOC-8**																																					
	Item 6			1.37			793			3.28			7.44			10.34			16.27			19.17			27.49			16.02			0.79				0.40		
	Item 8			1.37			793			3.28			7.06			12.11			19.55			15.51			25.35			17.15			0.78				0.43		
	Item 9			2.11			787			2.03			5.84			11.56			18.17			14.49			25.54			22.36			0.80				0.37		
	Item 11			2.11			787			3.30			8.51			12.33			22.24			19.44			23.38			10.80			0.81				0.34		
	Item 10			1.87			789			3.17			11.15			15.72			17.49			13.94			20.66			17.87			0.79				0.41		
	Item 13			2.11			787			2.54			6.35			10.67			14.23			16.90			31.77			17.53			0.80				0.38		
	Item 4			1.37			793			2.02			1.77			2.27			13.11			20.81			33.80			26.23			0.81				0.33		
	Item 12			1.99			788			4.31			5.46			9.14			13.07			19.04			32.11			16.88			0.80				0.35		

^a^Cronbach α is calculated if the item is removed; for example, if item 2 of SOC-13 were removed, the Cronbach α for comprehensibility (ie, Dimension 1) would increase from 0.64 to 0.71.

**Table 2:** Confirmatory factor analysis for the French versions of the 3-dimension 13-item and unidimensional 8-item sense of coherence (SOC) scales, and the correlation matrix for convergent and divergent validity for the French version of the 3-dimension scale.

Scales and items				Factor loading (SE)		Intercept (SE)		Error variance		Correlation matrix				
										Dimension 1		Dimension 2		Dimension 3
**SOC-13^a^**														
	**Dimension 1: comprehensibility (variance=0.06)**													
		Item 2	1.00		3.37 (0.05)		1.95		0.124		0.320		0.105	
		Item 6	4.29 (0.95)		4.87 (0.06)		1.49		0.520		0.517		0.377	
		Item 8	4.70 (1.04)		4.82 (0.06)		1.32		0.562		0.552		0.385	
		Item 9	3.71 (0.84)		5.03 (0.06)		1.76		0.476		0.415		0.298	
		Item 11	3.28 (0.75)		4.59 (0.06)		1.80		0.361		0.422		0.277	
	**Dimension 2: manageability (variance=0.17)**													
		Item 3	1.00		3.56 (0.06)		2.23		0.340		0.253		0.164	
		Item 5	1.77 (0.28)		4.83 (0.06)		2.10		0.397		0.339		0.265	
		Item 10	2.73 (0.40)		4.62 (0.06)		1.80		0.566		0.415		0.340	
		Item 13	2.29 (0.34)		5.03 (0.06)		1.65		0.516		0.390		0.324	
	**Dimension 3: meaningfulness (variance=0.02)**													
		Item 1	1.00		4.97 (0.06)		3.06		0.047		0.072		0.087	
		Item 4	5.72 (3.13)		5.55 (0.05)		1.17		0.383		0.305		0.324	
		Item 7	4.27 (2.34)		5.07 (0.05)		1.62		0.289		0.250		0.299	
		Item 12	7.51 (4.10)		5.00 (0.06)		1.63		0.430		0.434		0.317	
**SOC-8^b^ (variance=1.13)**														
	Item 6		1.00		4.87 (0.06)		1.54		—^c^		—		—	
	Item 8		1.12 (0.07)		4.82 (0.06)		1.32		—		—		—	
	Item 9		0.91 (0.07)		5.03 (0.06)		1.71		—		—		—	
	Item 11		0.79 (0.06)		4.58 (0.06)		1.80		—		—		—	
	Item 10		1.09 (0.07)		4.62 (0.06)		1.73		—		—		—	
	Item 13		0.91 (0.06)		5.02 (0.06)		1.61		—		—		—	
	Item 4		0.63 (0.05)		5.55 (0.05)		1.31		—		—		—	
	Item 12		0.84 (0.07)		5.01 (0.06)		1.87		—		—		—	

^a^SOC-13 scale: χ^2^_62_=436.36, χ^2^/*df*=7.0, root mean square error of approximation=0.088, standardized root mean square residual=0.067, comparative fit index=0.831; convergent validity: 4/13 items (30.8%) have a correlation coefficient with the score of their own dimension greater than 0.400; divergent validity: 5/13 items (38.5%) have a correlation coefficient with the score of their own dimension greater than those computed with other scores.

^b^Unidimensional SOC-8 scale: χ^2^_20_=61.90, χ^2^/*df*=3.1, root mean square error of approximation=0.052, standardized root mean square residual=0.030, comparative fit index=0.973.

^c^Not applicable; the dimensions are only relevant to the SOC-13 scale.

The results were similar for the unidimensional SOC-13, except that Cronbach α was higher than that found for the 3-dimension model (Cronbach α=0.79, Loevinger H=0.24, RMSEA=0.09, CFI=0.82). By contrast, the psychometric properties of the unidimensional SOC-8 were excellent, as shown in Tables 1 and 2 (Cronbach α=0.82, Loevinger H=0.38, RMSEA=0.05, CFI=0.97). The median score was 41 (IQR 13, range 8-56). The 3-dimension SOC-8 produced similar results, except that the Cronbach α values were lower (0.71 for comprehensibility, 0.55 for manageability, and 0.50 for meaningfulness; Loevinger H=0.39, 0.39, and 0.36, respectively; RMSEA=0.06; CFI=0.98).

## Discussion

We assessed the psychometric properties of the SOC scale within the framework of classical test theory. In a French primary care patient population, the validity/reliability of the French version of the 3-dimesion and unidimensional SOC-13 scale were poor. By contrast, the psychometric properties of the unidimensional SOC-8 were excellent. The properties of the 3-dimension SOC-8 scale were not better than those of the unidimensional model.

Despite the lack of validation studies (the general population study published in 2001 was based on a version of the questionnaire modified by the authors [6]), the SOC-13 has already been used in several studies in French-speaking populations, including in France [13] and Belgium [14]. However, our study findings indicate that the French questionnaire lacks validity and reliability, possibly influenced by language-specific nuances affecting the scale’s psychometric properties.

Researchers interested in assessing the SOC could perhaps use the unidimensional SOC-8 in the future, which showed excellent psychometric properties in our study, although further validation studies are still needed. All three dimensions (comprehensibility, meaningfulness, and manageability) are represented in the 8 questions of the SOC-8. This confirms that they adequately represent the SOC in reality. The difference in psychometric properties between the SOC-8 and the SOC-13 can be explained by the fact that the 5 items excluded in the SOC-8 are perhaps less clear in French and could potentially lead to different interpretations among respondents.

Our study has several limitations. As this study was limited to patients in France, it raises questions about generalizability to other French-speaking populations. Additionally, reproducibility was not assessed and external validation of the SOC-8 is crucial before widespread adoption.

In conclusion, our study suggests that the psychometric properties of the French version of the 3-dimension SOC-13 are poor, unlike the unidimensional SOC-8. A questionnaire built only with these 8 items could be a good candidate to measure SOC. However, further validation studies are needed before recommending its use in research.

## Appendix

Multimedia Appendix 1 The French versions of the SOC-13 and the SOC-8.

## Abbreviations

CFI: comparative fit index
RMSEA: root mean square error of approximation
SOC: sense of coherence
SOC-8: 8-item sense of coherence scale
SOC-13: 13-item sense of coherence scale

## Data Availability

The data associated with this article are available in the Open Science Framework [15].
