# Supplementary material for: Validation and Refinement of the Sense of Coherence Scale for a French Population: Observational Study
Source: Interact J Med Res. 2024 Jul 16;13:e50284. doi: 10.2196/50284 (PMC11289574; doi:10.2196/50284)
Supplement: Multimedia Appendix 1 [file ijmr_v13i1e50284_app1.docx]

**French version of the SOC-13 questionnaire**

Pour chaque question, merci de cocher le numéro correspondant le mieux à ce que vous ressentez (sur une échelle de 1 à 7).

| **1)**  **Vous avez le sentiment que vous ne vous souciez pas réellement de ce qui se passe autour de vous** | | | | | | | | |
| --- | --- | --- | --- | --- | --- | --- | --- | --- |
| **Très rarement ou jamais** | 1  ☐ | 2  ☐ | 3  ☐ | 4  ☐ | 5  ☐ | 6  ☐ | 7  ☐ | **Très souvent** |
| **2)**  **Vous est-il arrivé dans le passé d’être surpris.e par le comportement de gens que vous pensiez connaître très bien ?** | | | | | | | | |
| **Jamais** | 1  ☐ | 2  ☐ | 3  ☐ | 4  ☐ | 5  ☐ | 6  ☐ | 7  ☐ | **Toujours** |
| **3)** **Est-il arrivé que des gens sur lesquels vous comptiez vous déçoivent ?** | | | | | | | | |
| **Jamais** | 1  ☐ | 2  ☐ | 3  ☐ | 4  ☐ | 5  ☐ | 6  ☐ | 7  ☐ | **Toujours** |
| **4)** **Jusqu’à maintenant, votre vie n’a eu :** | | | | | | | | |
| **Aucun but ni objectif** | 1  ☐ | 2  ☐ | 3  ☐ | 4  ☐ | 5  ☐ | 6  ☐ | 7  ☐ | **Des buts et des objectifs très clairs** |
| **5)** **Avez-vous le sentiment que vous êtes traité.e injustement ?** | | | | | | | | |
| **Très souvent** | 1  ☐ | 2  ☐ | 3  ☐ | 4  ☐ | 5  ☐ | 6  ☐ | 7  ☐ | **Très rarement ou jamais** |
| **6)** **Avez-vous le sentiment que vous êtes dans une situation inconnue et que vous ne savez pas quoi faire ?** | | | | | | | | |
| **Très souvent** | 1  ☐ | 2  ☐ | 3  ☐ | 4  ☐ | 5  ☐ | 6  ☐ | 7  ☐ | **Très rarement ou jamais** |
| **7)** **Faire les choses que vous faites quotidiennement est une source :** | | | | | | | | |
| **De plaisir et de satisfaction profonde** | 1  ☐ | 2  ☐ | 3  ☐ | 4  ☐ | 5  ☐ | 6  ☐ | 7  ☐ | **De souffrance et d’ennui** |
| **8)** **Avez-vous des idées ou des sentiments confus ?** | | | | | | | | |
| **Très souvent** | 1  ☐ | 2  ☐ | 3  ☐ | 4  ☐ | 5  ☐ | 6  ☐ | 7  ☐ | **Très rarement ou jamais** |

| **9) Vous arrive-t-il d’avoir des sentiments intimes que vous préféreriez ne pas avoir ?** | | | | | | | | |
| --- | --- | --- | --- | --- | --- | --- | --- | --- |
| **Très souvent** | 1  ☐ | 2 ☐ | 3  ☐ | 4  ☐ | 5  ☐ | 6  ☐ | 7  ☐ | **Très rarement ou jamais** |
| **10) Beaucoup de gens (même s’ils ont beaucoup de caractère) se sentent parfois de pauvres cloches. Avez-vous déjà eu ce sentiment dans le passé ?** | | | | | | | | |
| **Très souvent** | 1  ☐ | 2  ☐ | 3  ☐ | 4  ☐ | 5  ☐ | 6  ☐ | 7  ☐ | **Très rarement ou jamais** |
| **11) Quand quelque chose arrive, vous trouvez généralement que :** | | | | | | | | |
| **Vous surestimez ou sous-estimez son importance** | 1  ☐ | 2  ☐ | 3  ☐ | 4  ☐ | 5  ☐ | 6  ☐ | 7  ☐ | **Vous voyez les choses dans de justes proportions** |
| **12) Avez-vous le sentiment que les choses que vous faites dans la vie quotidienne ont peu de sens ?** | | | | | | | | |
| **Très souvent** | 1  ☐ | 2  ☐ | 3  ☐ | 4  ☐ | 5  ☐ | 6  ☐ | 7  ☐ | **Très rarement ou jamais** |
| **13) Vous avez le sentiment que vous n’êtes pas sûr.e de vous maîtriser** | | | | | | | | |
| **Très souvent** | 1  ☐ | 2  ☐ | 3  ☐ | 4  ☐ | 5  ☐ | 6  ☐ | 7  ☐ | **Très rarement ou jamais** |

**French-version of the SOC-8 questionnaire**

Pour chaque question, merci de cocher le numéro correspondant le mieux à ce que vous ressentez (sur une échelle de 1 à 7).

| **4)** **Jusqu’à maintenant, votre vie n’a eu :** | | | | | | | | |
| --- | --- | --- | --- | --- | --- | --- | --- | --- |
| **Aucun but ni objectif** | 1  ☐ | 2  ☐ | 3  ☐ | 4  ☐ | 5  ☐ | 6  ☐ | 7  ☐ | **Des buts et des objectifs très clairs** |
| **6)** **Avez-vous le sentiment que vous êtes dans une situation inconnue et que vous ne savez pas quoi faire ?** | | | | | | | | |
| **Très souvent** | 1  ☐ | 2  ☐ | 3  ☐ | 4  ☐ | 5  ☐ | 6  ☐ | 7  ☐ | **Très rarement ou jamais** |
| **8)** **Avez-vous des idées ou des sentiments confus ?** | | | | | | | | |
| **Très souvent** | 1  ☐ | 2  ☐ | 3  ☐ | 4  ☐ | 5  ☐ | 6  ☐ | 7  ☐ | **Très rarement ou jamais** |

| **9) Vous arrive-t-il d’avoir des sentiments intimes que vous préféreriez ne pas avoir ?** | | | | | | | | |
| --- | --- | --- | --- | --- | --- | --- | --- | --- |
| **Très souvent** | 1  ☐ | 2 ☐ | 3  ☐ | 4  ☐ | 5  ☐ | 6  ☐ | 7  ☐ | **Très rarement ou jamais** |
| **10) Beaucoup de gens (même s’ils ont beaucoup de caractère) se sentent parfois de pauvres cloches. Avez-vous déjà eu ce sentiment dans le passé ?** | | | | | | | | |
| **Très souvent** | 1  ☐ | 2  ☐ | 3  ☐ | 4  ☐ | 5  ☐ | 6  ☐ | 7  ☐ | **Très rarement ou jamais** |
| **11) Quand quelque chose arrive, vous trouvez généralement que :** | | | | | | | | |
| **Vous surestimez ou sous-estimez son importance** | 1  ☐ | 2  ☐ | 3  ☐ | 4  ☐ | 5  ☐ | 6  ☐ | 7  ☐ | **Vous voyez les choses dans de justes proportions** |
| **12) Avez-vous le sentiment que les choses que vous faites dans la vie quotidienne ont peu de sens ?** | | | | | | | | |
| **Très souvent** | 1  ☐ | 2  ☐ | 3  ☐ | 4  ☐ | 5  ☐ | 6  ☐ | 7  ☐ | **Très rarement ou jamais** |
| **13) Vous avez le sentiment que vous n’êtes pas sûr.e de vous maîtriser** | | | | | | | | |
| **Très souvent** | 1  ☐ | 2  ☐ | 3  ☐ | 4  ☐ | 5  ☐ | 6  ☐ | 7  ☐ | **Très rarement ou jamais** |
